# SINE Insertion in *LAMA3* in Dogs With Junctional Epidermolysis Bullosa

**DOI:** 10.1002/age.70190

**Published:** 2026-08-23

**Authors:** Sarah Kiener, Ronnie Kaufmann, Ori Brenner, Vidhya Jagannathan, Tosso Leeb

**Affiliations:** ^1^ Vetsuisse Faculty, Institute of Genetics University of Bern Bern Switzerland; ^2^ Veterinary Teaching Hospital, The Koret School of Veterinary Medicine, The Hebrew University of Jerusalem Rehovot Israel; ^3^ Department of Veterinary Resources Weizmann Institute of Science Rehovot Israel

**Keywords:** *Canis lupus familiaris*, genodermatosis, laminin, skin, whole genome sequencing

## Abstract

Junctional epidermolysis bullosa (JEB) is a hereditary skin disorder caused by defects in proteins responsible for dermal‐epidermal adhesion. We investigated the genetic cause of JEB in three related mixed‐breed puppies presenting with congenital skin blistering and ulceration. Whole‐genome sequencing of one affected dog followed by comparison with 1538 control genomes identified a private candidate variant, XM_038543644.1:c.5690_5691ins240, in *LAMA3*, a known JEB‐associated gene. Visual inspection of the short‐read alignments and Sanger sequencing revealed a homozygous 240‐bp SINE insertion in exon 45 that had initially been miscalled as a heterozygous short insertion. Fragment length analysis confirmed complete co‐segregation of the variant with the disease phenotype within the available family. The SINE insertion is flanked by a 16‐bp target site duplication, contains a 45‐nt poly(A) tail, and is predicted to remain in‐frame, introducing an additional 80 amino acids into the laminin α3 coiled‐coil domain without introducing a premature stop codon. Although the molecular consequences were not functionally investigated, the insertion is expected to disrupt normal laminin‐332 heterotrimer assembly and secretion. This study expands the spectrum of pathogenic *LAMA3* variants associated with canine JEB and highlights the importance of visual inspection of short‐read sequencing data for the detection and correct interpretation of structurally complex variants such as transposable element insertions.

## 1 Introduction

Healthy skin forms the external boundary between the organism and the environment and is very resistant to mechanical stress. Key structures for this resilience are intermediate filaments in the basal keratinocytes, the hemidesmosomes at the dermal‐epidermal junction, and the anchoring fibrils in the basement membrane zone (Uitto and Richard 2005). Defects in any of these structures can result in epidermolysis bullosa (EB), a group of inherited blistering disorders characterized by dermo‐epidermal tissue separation following minimal trauma. Based on the level of skin cleavage, EB is classified into four major types: EB simplex, junctional EB (JEB), dystrophic EB, and Kindler EB. To date, pathogenic variants in at least 16 genes have been associated with the different forms of EB in humans (Bardhan et al. 2020; Has et al. 2020).

JEB results from defects in proteins that mediate adhesion between basal keratinocytes and the underlying basement membrane. Laminin‐332, composed of the laminin α3, β3, and γ2 chains, is a key structural component of the basement membrane that anchors the epidermis to the dermis. Additional proteins involved in this adhesion complex include collagen XVII and the α6β4 integrin. Pathogenic variants affecting genes coding for these proteins result in separation at the dermal‐epidermal junction, the characteristic histopathological feature of JEB (Bardhan et al. 2020; McMillan et al. 2003).

Naturally occurring JEB has been reported in several domestic animal species, including dogs, cats, cattle, horses, and sheep. Molecular studies have identified pathogenic variants in several of the same genes implicated in human disease, namely *LAMA3* (Capt et al. 2005; Graves et al. 2009; Herrmann et al. 2021; Sartelet et al. 2015), *LAMB3* (Kiener et al. 2020; Letko et al. 2025), *LAMC2* (Mömke et al. 2011; Murgiano et al. 2015; Spirito et al. 2002), *COL17A1* (Fussell et al. 2026; Kiener et al. 2023), *ITGA6* (Boussaha et al. 2023), and *ITGB4* (Fabre et al. 2021; Peters et al. 2015; Suárez‐Vega et al. 2015). These findings demonstrate that the genetic basis of JEB is highly conserved across humans and domestic animal species (Medeiros and Riet‐Correa 2015).

In this study, we describe the clinical, histopathological, and molecular characterization of JEB in three random‐bred puppies. Using whole‐genome sequencing and targeted molecular analyses, we identified a homozygous exonic SINE insertion in *LAMA3* as the likely genetic cause of the disease.

## 2 Materials and Methods

### 2.1 Animals

Three random‐bred puppies with JEB were included in this study. In addition, the unaffected mother of two of the puppies and one clinically unaffected littermate were available for genetic analyses. A cohort of 1538 publicly available whole‐genome sequences from genetically diverse dogs was used for variant filtering (Table S1). One unrelated control dog was included for targeted genotyping.

### 2.2 Clinical and Histopathological Examination

All affected dogs underwent clinical examination. Skin biopsies were collected from lesional skin at several anatomical sites and routinely processed for histopathological examination. Histopathological evaluation was performed to confirm the diagnosis of epidermolysis bullosa and characterize the microscopic lesions.

### 2.3 Whole Genome Sequencing and Variant Calling

Genomic DNA from all dogs included in the study was isolated from EDTA blood with the Maxwell RSC Whole Blood DNA Kit using a Maxwell RSC instrument (Promega, Dübendorf, Switzerland). One of the affected female dogs was whole‐genome sequenced as follows. A PCR‐free library was prepared from genomic DNA using the Illumina TruSeq DNA PCR‐Free Library Prep Kit (Illumina, San Diego, CA, USA) following the manufacturer's protocol. The library was sequenced on an Illumina NovaSeq 6000 instrument (Illumina, San Diego, CA, USA) in paired‐end 2 × 150 bp mode (within a pool of other libraries on an S4 flow cell) at the Next Generation Sequencing Platform, University of Bern, Switzerland. The sequencing produced 258.3 million read pairs. Variant calling followed the GATK best‐practices workflow as implemented in our in‐house Nextflow DSL2 pipeline DogWGS (https://github.com/vetsuisse‐unibe/DogWGS). Per‐sample reads were adapter and quality‐trimmed with fastp [v0.23.4], aligned to a modified version of the German Shepherd Dog genome assembly (UU_Cfam_GSD_1.0, GCF_011100685.1; Wang et al. 2021), supplemented by three Y chromosome sequences from a Labrador Retriever (ROS_Cfam_1.0, GCF_014441545.1) with BWA‐MEM2 2.2.1. Alignments were coordinate‐sorted (SAMtools v1.13) and PCR/optical duplicates were marked with Picard MarkDuplicates (v2.21.8). After base quality score recalibration (BQSR), variants were called per sample with GATK v4.2.6.1 HaplotypeCaller (GVCF mode), jointly genotyped across samples (GenomicsDBImport → GenotypeGVCFs), filtered (VQSR/hard filtering) and annotated with snpEff (v5.0e). 99.91% of the sequenced reads mapped to the reference and 98.78% were properly paired, with a duplication rate of 5.70% and a mean mismatch rate of 0.63%. The deduplicated alignments covered 98.27% of the reference at ≥ 1× and showed a mean coverage of 29.3×. The genome was deposited at the European Nucleotide Archive with the accession number SAMEA10833662.

### 2.4 Variant Filtering

Starting from the multi‐sample, snpEff‐annotated cohort VCF comprising the affected dog and 1538 publicly available canine whole‐genome sequences (Table S1), snpEff annotations were flattened to one transcript effect per line, producing a tab‐delimited table of variant coordinates, alleles, per‐sample genotypes, and predicted functional consequences. As the mode of inheritance was unknown, both dominant and recessive disease models were considered. Candidate variants private to the affected dog were extracted using a custom pipeline. Variants were retained only where the case was heterozygous (0/1) or homozygous alternate (1/1) and all remaining animals were homozygous reference (0/0) or missing (./.). Private variants were subsequently filtered by retaining only protein‐changing variants, which were the variants with a SnpEff predicted impact of HIGH or MODERATE. Finally, the remaining variants were prioritized based on their presence in 16 known epidermolysis bullosa candidate genes (Has et al. 2020).

### 2.5 Variant Validation and Targeted Genotyping

The candidate variant *LAMA3*:XM_038543644.1:c.5690_5691ins240 was validated by Sanger sequencing and genotyped in all affected dogs, the available family members and one unrelated control dog by fragment length analysis. PCR amplification was performed using the QIAGEN Multiplex PCR Kit (Qiagen, Hilden, Germany) according to the manufacturer's instructions, with an annealing temperature of 60°C and 35 amplification cycles. The forward primer was 5′‐TCA TGG ATT AAA AAT GGA TGG TC‐3′ and the reverse primer was 5′‐TGC ATG GTT TCA AAT TCA CG‐3′. PCR products were treated with exonuclease I and alkaline phosphatase prior to fragment length analysis on a 5200 Fragment Analyzer (Agilent, Basel, Switzerland). Purified PCR products were additionally sequenced on an ABI 3730 DNA Analyzer (Thermo Fisher Scientific, Waltham, MA, USA) to determine the exact sequence of the inserted SINE element. Sequence chromatograms were analyzed using Sequencher 5.1 software (GeneCodes, Ann Arbor, MI, USA).

## 3 Results

### 3.1 Clinical and Histopathological Examination

**FIGURE 1:**
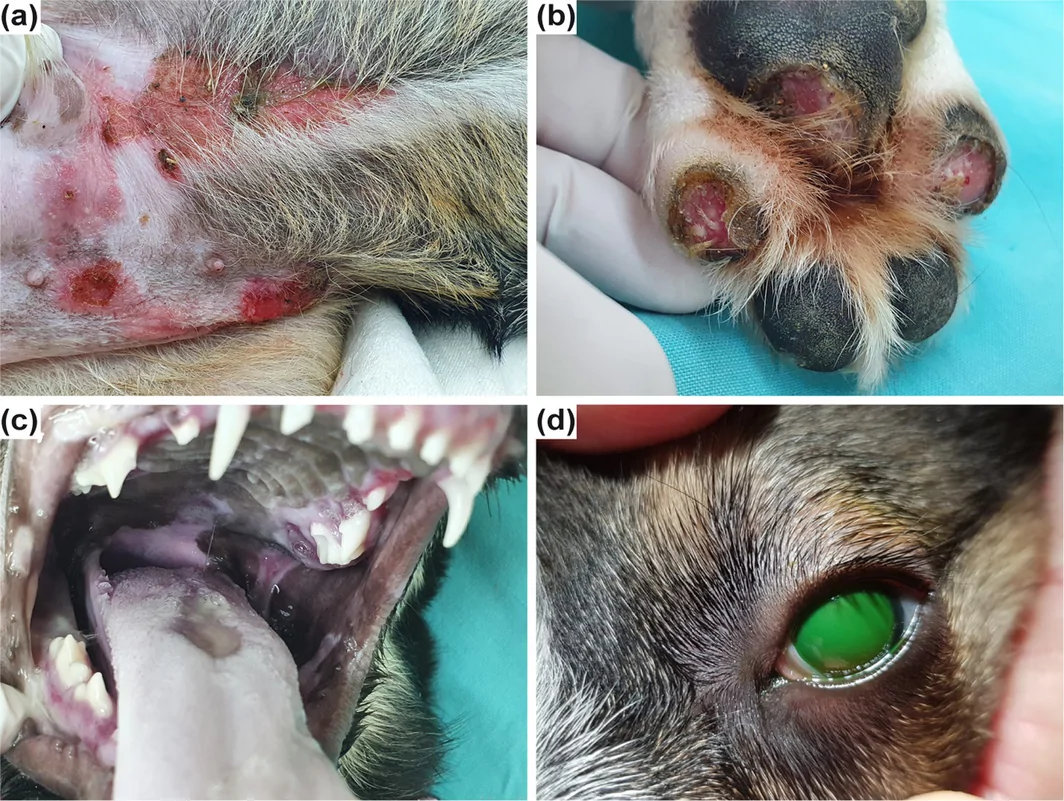
Clinical phenotype. (a) Multifocal ulcers in the abdominal area of an affected puppy. (b) Severe, deep paw‐pad ulcers. (c) Large ulcer on the dorsal aspect of the tongue (the picture was taken postmortem). (d) Large corneal ulcer. Fluorescein staining showed complete staining of the cornea.

Two female and one male random‐bred puppies were investigated. Two of the affected dogs were littermates, whereas the exact relationship to the third puppy was unknown. All three affected puppies shared similar clinical signs, which started at around 6–8 weeks of age and included complete loss of nails, deep ulcers in the paw pads and oral mucosa, vesicles and ulcers in the inner pinna, ulcers and bullae in the inguinal area and abdomen, and enamel hypoplasia (Figure 1a–c). Two of the puppies also suffered from unilateral non‐healing very large corneal ulcers (Figure 1d). All three affected puppies were euthanized a few weeks after diagnosis due to severe clinical deterioration, loss of appetite, and unmanageable pain.

**FIGURE 2:**
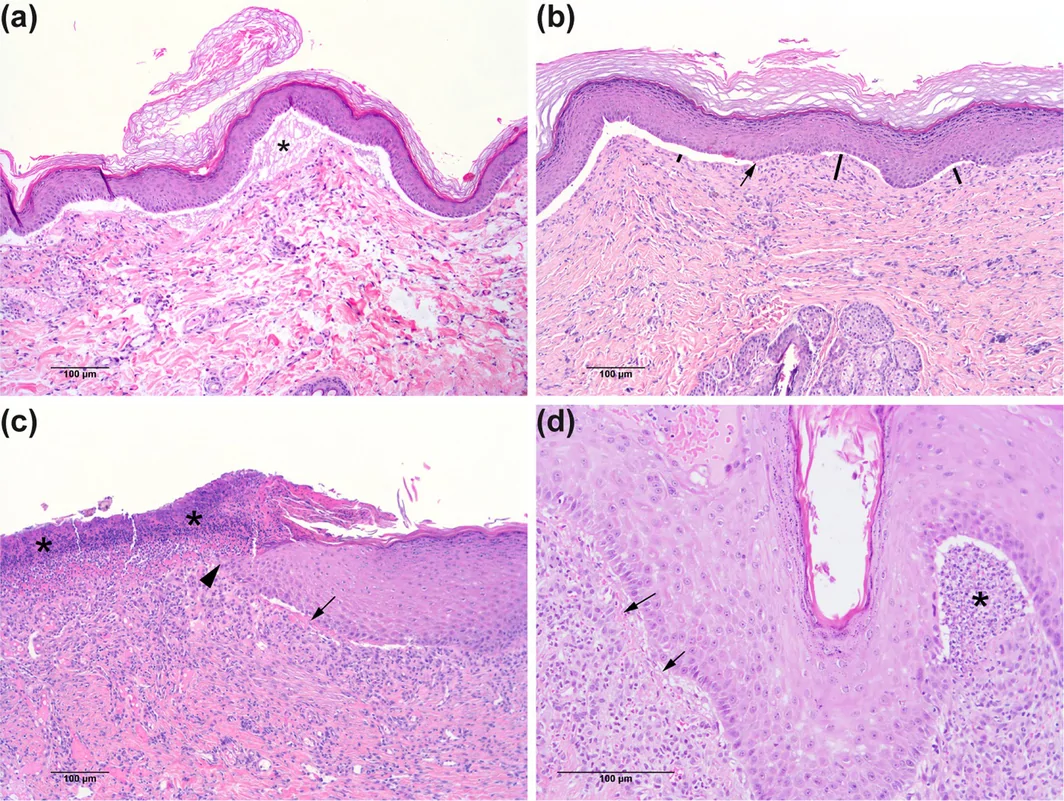
Histopathological findings in skin biopsies from affected puppies. (a) Acute lesion. The epidermis is detached from the dermis. A collection of fibrin (asterisk) is found in the vesicle. The changes in the dermis are mild. (b) Subacute lesion (without evidence of secondary bacterial infection). In the superficial dermis there is mild fibrosis (spanned by black lines). Arrow: a small amount of fibrin in the space created by dermo‐epidermal separation. (c) Advanced lesion with ulceration and evidence of secondary bacterial infection. There is marked epidermal hyperplasia. The arrowhead points to the growing tip of the epidermis, indicative of epidermal regeneration. To the left of the arrowhead, there is an ulcer covered by fibrinopurulent debris (asterisks). The arrow points to a small amount of fibrin in the space created by dermo‐epidermal separation. In the dermis there is widespread fibrosis and moderate inflammation. (d) Advanced lesion with evidence of secondary bacterial infection. The asterisk indicates a collection of neutrophils in a vesicle created by dermo‐epidermal separation. The arrows point to fibrin in a narrow space between markedly hyperplastic epidermis and dermis with significant fibrosis and inflammation.

Diagnosis of EB was confirmed by biopsies taken from lesional skin. The most consistent finding was dermo‐epidermal separation. It was accompanied by a range of reactive changes, which depended on the duration of the lesion and the presence of secondary bacterial infection. Lesions with different microscopic features were found near each other in most samples. In acute lesions, the epidermis was either of normal height or moderately hyperplastic and there was often accumulation of fibrin and/or blood in the space created by dermo‐epidermal separation (Figure 2a). Changes in the dermis consisted of vascular dilation, hypertrophy of fibroblasts and vascular cells, and mild neutrophilic infiltration. In subacute lesions, moderately hyperplastic epidermis was separated from dermis in which there was mild superficial fibrosis (Figure 2b). In longer‐standing lesions with evidence of secondary bacterial infection and/or recurrence, epidermal hyperplasia, dermal fibrosis and inflammation were more pronounced and there was ulceration, serocellular crusting, and epidermal regeneration (Figure 2c). Occasionally, neutrophils collected below the lifted epidermis (Figure 2d).

### 3.2 Genetic Analysis

**FIGURE 3:**
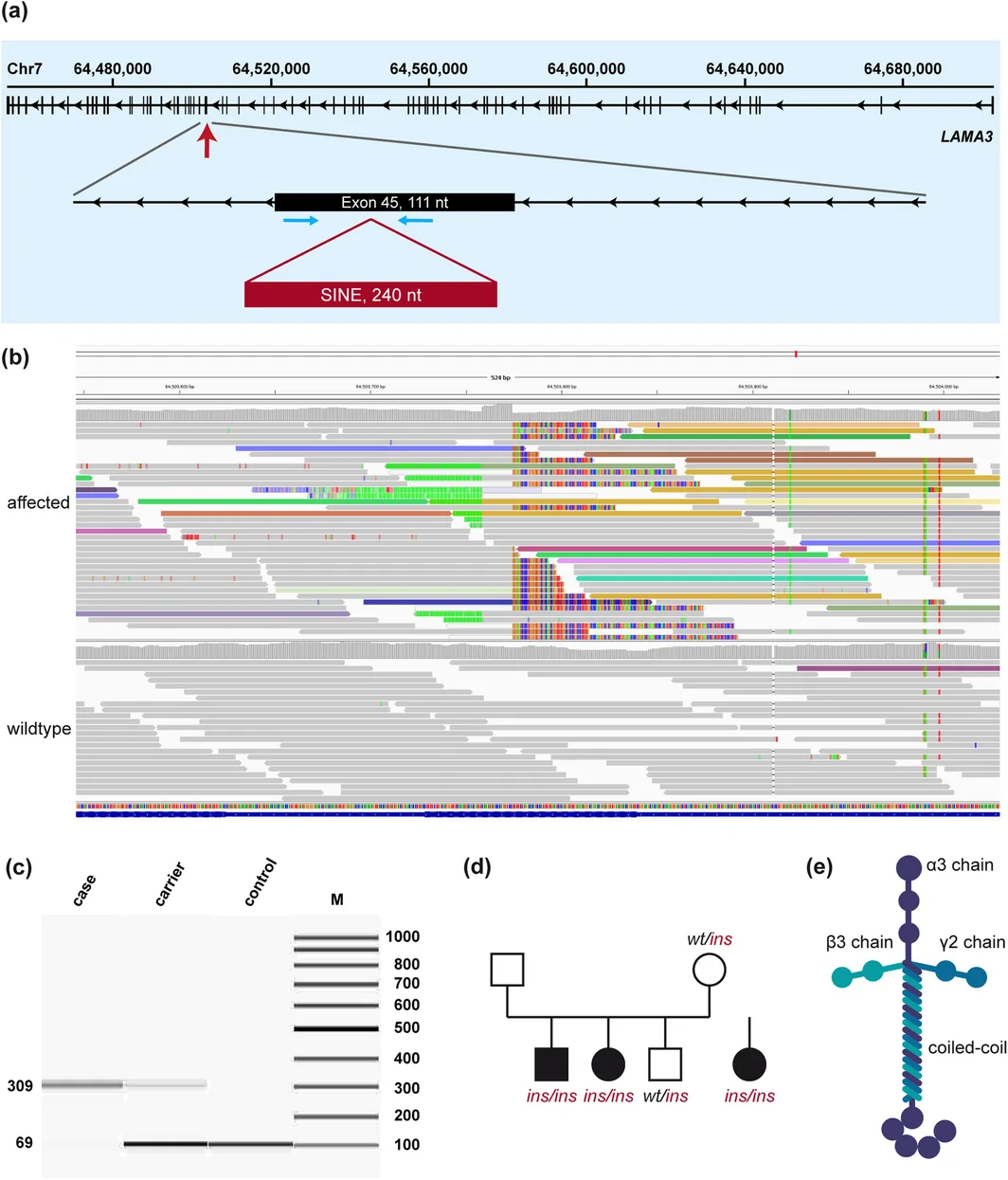
Details of the *LAMA3* variant. (a) Schematic representation of the genomic organization of the *LAMA3* gene showing introns and exons. The position of the identified variant, XM_038543644.1:c.5690_5691ins240, is indicated by an arrow. An enlarged view of exon 45 of 75 shows the position of the SINE insertion. The locations of the forward and reverse PCR primers used for genotyping are indicated by blue arrows. (b) Integrative Genomics Viewer (IGV) screenshot showing short‐read alignments from the affected dog and an unrelated control dog at the site of the SINE insertion. (c) Fragment length analysis of PCR amplicons from a JEB‐affected dog, an unaffected relative, and an unrelated healthy control dog, demonstrating the 240‐nucleotide insertion in the mutant allele. Brightness and contrast were uniformly adjusted for clarity. (d) Pedigree of the investigated random‐bred dogs. Squares represent males and circles represent females. Filled symbols indicate affected dogs. Genotypes for the *LAMA3*:c.5690_5691ins240 variant are shown below each individual for which DNA samples were available. (e) Schematic representation of the laminin‐332 heterotrimer. The complex consists of the α3, β3, and γ2 chains, with the coiled‐coil domain mediating heterotrimer assembly. Created with BioRender.com.

Comparison of the WGS data of the affected dog with 1538 publicly available canine genomes identified 214 heterozygous and 60 homozygous private protein‐changing variants (Table S2). Screening of 16 known EB candidate genes (Has et al. 2020), identified a single candidate variant in *LAMA3*. Although initially called as heterozygous, visual inspection of the short‐read alignments established that the variant was in fact a homozygous exonic SINE insertion into the *LAMA3* gene (XM_038543644.1:c.5690_5691ins240; Figure 3a,b).

Sanger sequencing confirmed a 240‐bp SINE insertion in exon 45 of *LAMA3* and determined its exact sequence and insertion site (File S1). The insertion was flanked by a 16‐bp target site duplication and contained a poly(A) tail. These two features were also evident from the short‐read alignments (Figure 3b and File S1). Sequence analysis showed that the insertion is in‐frame and predicted to introduce an additional 80 amino acids within the coiled‐coil domain of laminin α3 without introducing a premature termination codon (XP_038399572.1:p.(Lys1897_Glu1898insXaa[80])).

Fragment length analysis demonstrated complete concordance between genotype and phenotype in the investigated family. All three affected puppies were homozygous for the insertion, whereas their unaffected dam and the unaffected littermate were heterozygous carriers. The unrelated control dog was homozygous for the reference allele (Figure 3c,d).

## 4 Discussion

In this study, we identified a novel homozygous exonic SINE insertion in *LAMA3* as the likely cause of junctional epidermolysis bullosa (JEB) in three random‐bred puppies. The variant was absent from 1538 publicly available canine whole‐genome sequences and represented the only protein‐changing private variant in a known EB candidate gene identified in the WGS data of a JEB affected dog. Genotypes at the variant co‐segregated with the disease phenotype within the investigated family. Applying the ACMG/AMP criteria for the interpretation of sequence variants in human diagnostics (Richards et al. 2015) or the AVCG v2 criteria (Desmet et al. 2026), we classified the variant as pathogenic (Table S3).

*LAMA3* is a well‐studied candidate gene for JEB both in humans and animals (Capt et al. 2005; Graves et al. 2009; Has et al. 2020; Herrmann et al. 2021; Kivirikko et al. 1995; Sartelet et al. 2015). It encodes the laminin alpha 3 chain, one of the three subunits of laminin‐332, together with the β3 and γ2 chains encoded by *LAMB3* and *LAMC2*, respectively. Laminin‐332 is a crucial component of the basement membrane that anchors epithelial cells to the underlying tissues. Its assembly begins in the endoplasmic reticulum, where the β3 and γ2 chains first form a heterodimer before incorporation of the α3 chain completes the mature heterotrimer. Consequently, the α3 chain is essential for efficient laminin‐332 assembly and secretion, and pathogenic variants affecting any of the three subunits disrupt the integrity of the complex and compromise dermal–epidermal adhesion (Bardhan et al. 2020; Parzymies 2026; Sugawara et al. 2008).

Assuming that the SINE insertion does not alter pre‐mRNA splicing, which was not investigated in the present study, the insertion is predicted to maintain the open reading frame and to introduce an additional 80 amino acids into the coiled‐coil domain of laminin α3. The predicted insertion contains a stretch of 15 phenylalanines encoded by the poly(A) tail of the SINE insertion. Such an amino acid sequence would clearly not be compatible with the heptad repeat organization of a coiled‐coil domain (Lupas 1996). The coiled‐coil domain mediates heterotrimer formation with the β3 and γ2 chains and is therefore critical for normal laminin‐332 assembly (Domogatskaya et al. 2012). Although the functional consequences of the insertion remain to be determined, the substantial in‐frame expansion within this highly conserved structural domain is expected to interfere with normal coiled‐coil formation and thereby severely compromise laminin‐332 assembly and/or secretion.

The predicted functional consequence of the identified variant is consistent with the severe clinical phenotype observed in the affected puppies. In humans, severe generalized JEB is typically associated with biallelic loss‐of‐function variants in *LAMA3*, *LAMB3*, or *LAMC2*, whereas variants permitting residual laminin‐332 expression generally result in milder phenotypes (Parzymies 2026). Similarly, *Lama3* ^*−/−*^ null mice develop extensive skin blistering shortly after birth, exhibit abnormal hemidesmosomes, loss of anchorage function in the epidermis, and die within a few days of birth (Abrass et al. 2006; Yao 2017).

An interesting technical finding was that the causal variant was initially reported by GATK HaplotypeCaller as a heterozygous 53 bp insertion rather than a homozygous 240 bp SINE insertion. Visual inspection of the short‐read alignments revealed numerous soft‐clipped reads and discordant read pairs spanning the breakpoint, indicating the presence of a substantially larger insertion. As SINE elements are present in many copies throughout the canine genome, reads originating within the inserted element cannot be mapped uniquely and therefore receive low mapping‐quality scores, whereas reads spanning the unique flanking sequence align with high confidence. Filtering of low‐quality reads by the variant caller resulted in an artificial allelic imbalance, leading to an erroneous heterozygous genotype call. This observation illustrates a limitation of standard short‐read variant‐calling pipelines for repetitive element insertions and highlights the importance of visual inspection of candidate variants, particularly when evaluating plausible disease‐causing structural variants.

Although random‐bred dogs are often assumed to be genetically diverse, they can have high levels of inbreeding due to close matings such as between siblings or between parents and offspring (Leeb et al. 2023). The observed segregation pattern in this study is consistent with autosomal recessive inheritance and illustrates that inherited genetic disorders also occur in such populations, highlighting the importance of considering genetic diseases also in non‐pedigreed animals. It further expands the spectrum of pathogenic *LAMA3* variants causing canine JEB and demonstrates the value of whole‐genome sequencing combined with careful interpretation of structural variants for the diagnosis of inherited skin disorders in veterinary patients.

## Author Contributions

**Sarah Kiener:** investigation, visualization, writing – original draft, writing – review and editing. **Ronnie Kaufmann:** conceptualization, investigation, visualization, writing – original draft, writing – review and editing. **Tosso Leeb:** conceptualization, visualization, writing – original draft, writing – review and editing, funding acquisition. **Ori Brenner:** conceptualization, investigation, visualization, writing – original draft, writing – review and editing. **Vidhya Jagannathan:** data curation, writing – review and editing.

## Funding

This research was funded by the Swiss National Science Foundation (Grant 310030_200354).

## Ethics Statement

All examinations and animal experiments were carried out after obtaining written informed owner's consent and in accordance with local laws, regulations, and ethical guidelines. Dog blood samples were collected with the approval of the Cantonal Committee for Animal Experiments (Canton of Bern, Switzerland; permits BE 71/19 & BE94/2022). Skin punch biopsies were taken for diagnostic purposes with informed consent of the owners, in accordance with ethical guidelines and approved procedures.

## Conflicts of Interest

The authors declare no conflicts of interest.

## Supporting information


**File S1:** Sequence context of the 240 bp SINE insertion into exon 45 of the canine LAMA3 gene.

**Table S1:** Accession numbers of 1539 dog genome sequences. The affected dog is highlighted in red. The other 1538 genome sequences were used as controls in variant filtering.

**Table S2:** Private variants in the sequenced dog affected with junctional epidermolysis bullosa. Private variants were obtained by filtering all variants that were present at least once in the 1538 control genomes. This resulted in 30 854 heterozygous private variants for the affected dog. Please note that variants are listed multiple times, if they have predicted effects on more than one transcript. The LAMA3:c.5690_5691ins240 variant was incorrectly called as a heterozygous variant. It is highlighted in yellow in line 21 288 of this.

**Table S3:** Classification of the LAMA3:XM_038543644.1:c.5690_5691ins240 variant.

## Data Availability

The genome sequence data were submitted to the European Nucleotide Archive (ENA). All accession numbers are listed in Table S1.

## References

[age70190-bib-0001] Abrass, C. K. , A. K. Berfield , M. C. Ryan , W. G. Carter , and K. M. Hansen . 2006. “Abnormal Development of Glomerular Endothelial and Mesangial Cells in Mice With Targeted Disruption of the *LAMA3* Gene.” Kidney International 70: 1062–1071. 16850021 10.1038/sj.ki.5001706

[age70190-bib-0002] Bardhan, A. , L. Bruckner‐Tuderman , I. L. C. Chapple , et al. 2020. “Epidermolysis Bullosa.” Nature Reviews Disease Primers 6: 78. 10.1038/s41572-020-0210-0 32973163

[age70190-bib-0003] Boussaha, M. , A. Boulling , V. Wolgust , et al. 2023. “Integrin Alpha 6 Homozygous Splice‐Site Mutation Causes a New Form of Junctional Epidermolysis Bullosa in Charolais Cattle.” Genetics Selection Evolution 55: 40. 10.1186/s12711-023-00814-1 PMC10258996 37308849

[age70190-bib-0004] Capt, A. , F. Spirito , E. Guaguere , A. Spadafora , J.‐P. Ortonne , and G. Meneguzzi . 2005. “Inherited Junctional Epidermolysis Bullosa in the German Pointer: Establishment of a Large Animal Model.” Journal of Investigative Dermatology 124: 530–535. 15737193 10.1111/j.0022-202X.2004.23584.x

[age70190-bib-0005] Desmet, L. , C. Ferrari , M. Abitbol , et al. 2026. “The Animal Variant Classification Guidelines v2: An Update With New Criteria and Improved Clarifications.” Animal Genetics. Submitted. 10.1002/age.70197 PMC13545154 42698066

[age70190-bib-0006] Domogatskaya, A. , S. Rodin , and K. Tryggvason . 2012. “Functional Diversity of Laminins.” Annual Review of Cell and Developmental Biology 28: 523–553. 10.1146/annurev-cellbio-101011-155750 23057746

[age70190-bib-0007] Fabre, S. , L. Chantepie , F. Plisson‐Petit , et al. 2021. “A Novel Homozygous Nonsense Mutation in *ITGB4* Gene Causes Epidermolysis Bullosa in Mouton Vendéen Sheep.” Animal Genetics 52: 138–139. 33225458 10.1111/age.13026

[age70190-bib-0008] Fussell, D. , M. Leber , M. W. Vandewege , et al. 2026. “Novel Frameshift Variant in Exon 7 of *COL17A1* in a Domestic Shorthair Kitten With Junctional Epidermolysis Bullosa.” Journal of Veterinary Diagnostic Investigation 38: 523–527. 41588668 10.1177/10406387251414540 PMC12846888

[age70190-bib-0009] Graves, K. T. , P. J. Henney , and R. B. Ennis . 2009. “Partial Deletion of the *LAMA3* Gene Is Responsible for Hereditary Junctional Epidermolysis Bullosa in the American Saddlebred Horse.” Animal Genetics 40: 35–41. 19016681 10.1111/j.1365-2052.2008.01795.x

[age70190-bib-0010] Has, C. , J. W. Bauer , C. Bodemer , et al. 2020. “Consensus Reclassification of Inherited Epidermolysis Bullosa and Other Disorders With Skin Fragility.” British Journal of Dermatology 183: 614–627. 32017015 10.1111/bjd.18921

[age70190-bib-0011] Herrmann, I. , K. E. Linder , K. M. Meurs , et al. 2021. “Canine Junctional Epidermolysis Bullosa due to a Novel Mutation in LAMA3 With Severe Upper Respiratory Involvement.” Veterinary Dermatology 32: 379–e108. 34250689 10.1111/vde.12972 PMC8279226

[age70190-bib-0012] Kiener, S. , A. Laprais , E. A. Mauldin , V. Jagannathan , T. Olivry , and T. Leeb . 2020. “ *LAMB3* Missense Variant in Australian Shepherd Dogs With Junctional Epidermolysis Bullosa.” Genes 11: 1055. 32906717 10.3390/genes11091055 PMC7565164

[age70190-bib-0013] Kiener, S. , H. Troyer , D. Ruvolo , et al. 2023. “Independent *COL17A1* Variants in Cats With Junctional Epidermolysis Bullosa.” Genes (Basel) 14: 1835. 37895184 10.3390/genes14101835 PMC10606533

[age70190-bib-0014] Kivirikko, S. , J. A. McGrath , C. Baudoin , et al. 1995. “A Homozygous Nonsense Mutation in the Alpha 3 Chain Gene of Laminin 5 (LAMA3) in Lethal (Herlitz) Junctional Epidermolysis Bullosa.” Human Molecular Genetics 4: 959–962. 7633458 10.1093/hmg/4.5.959

[age70190-bib-0015] Leeb, T. , D. Bannasch , and J. J. Schoenebeck . 2023. “Identification of Genetic Risk Factors for Monogenic and Complex Canine Diseases.” Annual Review of Animal Biosciences 11: 183–205. 36322969 10.1146/annurev-animal-050622-055534

[age70190-bib-0016] Letko, A. , L. Harkema , K. Peterson , R. Dijkman , and C. Drögemüller . 2025. “A Homozygous *LAMB3* Frameshift Variant in Junctional Epidermolysis Bullosa‐Affected Bleu du Maine Sheep.” Journal of Applied Genetics 66: 709–714. 40100311 10.1007/s13353-025-00957-5 PMC12367931

[age70190-bib-0017] Lupas, A. 1996. “Coiled Coils: New Structures and New Functions.” Trends in Biochemical Sciences 21: 375–382. 8918191

[age70190-bib-0018] McMillan, J. R. , M. Akiyama , and H. Shimizu . 2003. “Epidermal Basement Membrane Zone Components: Ultrastructural Distribution and Molecular Interactions.” Journal of Dermatological Science 31: 169–177. 12727020 10.1016/s0923-1811(03)00045-8

[age70190-bib-0019] Medeiros, G. X. , and F. Riet‐Correa . 2015. “Epidermolysis Bullosa in Animals: A Review.” Veterinary Dermatology 26: 3–13,e1‐2. 25354580 10.1111/vde.12176

[age70190-bib-0020] Mömke, S. , A. Kerkmann , A. Wöhlke , et al. 2011. “A Frameshift Mutation Within *LAMC2* Is Responsible for Herlitz Type Junctional Epidermolysis Bullosa (HJEB) in Black Headed Mutton Sheep.” PLoS One 6: e18943. 21573221 10.1371/journal.pone.0018943 PMC3087721

[age70190-bib-0021] Murgiano, L. , N. Wiedemar , V. Jagannathan , L. K. Isling , C. Drögemüller , and J. S. Agerholm . 2015. “Epidermolysis Bullosa in Danish Hereford Calves Is Caused by a Deletion in *LAMC2* Gene.” BMC Veterinary Research 11: 23. 25888738 10.1186/s12917-015-0334-8 PMC4328060

[age70190-bib-0022] Parzymies, P. 2026. “So Much More Than Just Structural Support: Laminin‐332 and Its Multifaceted Functions in Skin Homeostasis and Disease.” Experimental Dermatology 35: e70293. 42265945 10.1111/exd.70293

[age70190-bib-0023] Peters, M. , I. Reber , V. Jagannathan , B. Raddatz , P. Wohlsein , and C. Drögemüller . 2015. “DNA‐Based Diagnosis of Rare Diseases in Veterinary Medicine: A 4.4 kb Deletion of *ITGB4* Is Associated With Epidermolysis Bullosa in Charolais Cattle.” BMC Veterinary Research 11: 48. 25890340 10.1186/s12917-015-0366-0 PMC4351973

[age70190-bib-0024] Richards, S. , N. Aziz , S. Bale , et al. 2015. “Standards and Guidelines for the Interpretation of Sequence Variants: A Joint Consensus Recommendation of the American College of Medical Genetics and Genomics and the Association for Molecular Pathology.” Genetics in Medicine 17: 405–424. 25741868 10.1038/gim.2015.30 PMC4544753

[age70190-bib-0025] Sartelet, A. , C. Harland , N. Tamma , et al. 2015. “A Stop‐Gain in the Laminin, Alpha 3 Gene Causes Recessive Junctional Epidermolysis Bullosa in Belgian Blue Cattle.” Animal Genetics 46: 566–570. 26370913 10.1111/age.12342

[age70190-bib-0026] Spirito, F. , A. Charlesworth , J.‐P. Ortonne , G. Meneguzzi , K. Linder , and J. Baird . 2002. “Animal Models for Skin Blistering Conditions: Absence of Laminin 5 Causes Hereditary Junctional Mechanobullous Disease in the Belgian Horse.” Journal of Investigative Dermatology 119: 684–691. 12230513 10.1046/j.1523-1747.2002.01852.x

[age70190-bib-0027] Suárez‐Vega, A. , B. Gutiérrez‐Gil , J. Benavides , et al. 2015. “Combining GWAS and RNA‐Seq Approaches for Detection of the Causal Mutation for Hereditary Junctional Epidermolysis Bullosa in Sheep.” PLoS One 10: e0126416. 25955497 10.1371/journal.pone.0126416 PMC4425408

[age70190-bib-0028] Sugawara, K. , D. Tsuruta , M. Ishii , J. C. R. Jones , and H. Kobayashi . 2008. “Laminin‐332 and ‐511 in Skin.” Experimental Dermatology 17: 473–480. 18474082 10.1111/j.1600-0625.2008.00721.x

[age70190-bib-0029] Uitto, J. , and G. Richard . 2005. “Progress in Epidermolysis Bullosa: From Eponyms to Molecular Genetic Classification.” Clinical Dermatology 23: 33–40. 10.1016/j.clindermatol.2004.09.015 15708287

[age70190-bib-0030] Wang, C. , O. Wallerman , M. L. Arendt , et al. 2021. “A Novel Canine Reference Genome Resolves Genomic Architecture and Uncovers Transcript Complexity.” Communications Biology 4: 185. 33568770 10.1038/s42003-021-01698-x PMC7875987

[age70190-bib-0031] Yao, Y. 2017. “Laminin: Loss‐Of‐Function Studies.” Cellular and Molecular Life Sciences 74: 1095–1115. 27696112 10.1007/s00018-016-2381-0 PMC11107706

