# Supplementary material for: SINE Insertion in LAMA3 in Dogs With Junctional Epidermolysis Bullosa
Source: Anim Genet. 2026 Aug 23;57(5):e70190. doi: 10.1002/age.70190 (PMC13500218; doi:10.1002/age.70190)
Supplement: Supplementary file 1 [file AGE-57-0-s003.pdf]

File S1. Sequence context of the 240 bp SINE insertion into exon 45 of the canine *LAMA3* gene

Genetic variant: Chr7:NC_049228.1:g.64,503,774_64,503,775ins240
LAMA3:XM_038543644.1:c.5690_5691ins240

The sequence of UU_Cfam_GSD_1.0 assembly, chromosome 7 genomic sequence, positions 64,503,701 - 64,503,880 (NC_049228.1) is indicated below.

The 111 nt sequence of *LAMA3* exon 45 (of 75 exons total) is given in black CAPITAL LETTERS. Note that the coding sequence of *LAMA3* is in reverse complementary orientation. Lowercase letters indicate intronic bases.

The sequence of the 240 bp SINE-insertion is given in blue.

16 bp duplicated sequence flanking the insertion site are underlined.

```
64,503,701      agttaccatt attgactgaa caccttacCT TTTCTTGCAT GGTTTCAAAT TCACGATTCA     64,503,760
64,503,761      AATTACCCAG TTCC                                                       64,503,774
                16 nt duplication flanking the insertion

64,503,774.1    GGGATCCCTG GGTGGCGCAG CGGTTTGGCG CCTGCCTTTG GCCCAGGGCG CGATCCTGGA     64,503,774.60
64,503,774.61   GACCCGGGAT CGAATCCCAC GTCGGGCTCC CGGTGCATGG AGCCTGCTTC TCCCTCTGCC     64,503,774.120
64,503,774.121  TGTGTCTCTG CCTCTCTCTC TCTCTCTGTG ACTATCATGA ATAAATAAAT AAAATCTTTA     64,503,774.180
64,503,774.181  AAAAAAAAAA AAAAAAAAAA AAAAAAAAAA AAAAAAAAAA AAAACAAATT ACCCAGTTCC     64,503,774.240
                                          16 nt duplication flanking the insertion

64,503,775                   TTTTCT AGACCATCCA TTTTTAATCC ATGATTTGAA ATGGCAGAAC     64,503,820
64,503,821     GATAGTTGAA TAACTGATTc tggaagcaca atgcaaagtg aatctcacat tagagcagga     64,503,880
```
